# Proinflammatory IFNγ Is Produced by but Not Required for the Generation of Eomes^+^ Thymic Innate CD8 T Cells

**DOI:** 10.3390/cells12202433

**Published:** 2023-10-11

**Authors:** Hee Yeun Won, Nurcin Liman, Can Li, Jung-Hyun Park

**Affiliations:** Experimental Immunology Branch, Center for Cancer Research, National Cancer Institute, NIH, Bethesda, MD 20892, USA; heeyeun.won@nih.gov (H.Y.W.); nora.liman@nih.gov (N.L.); can.li@nih.gov (C.L.)

**Keywords:** eomesodermin, IFNγ, IL-2Rβ, *i*NKT cells, thymus

## Abstract

Innate CD8 T cells are proinflammatory effector T cells that achieve functional maturation in the thymus prior to their export into and maturation in peripheral tissues. Innate CD8 T cells produce the Th1 cytokine IFNγ but depend on the Th2 cytokine IL-4 for their generation. Thus, innate CD8 T cells can permute the intrathymic cytokine milieu by consuming a Th2 cytokine but driving a Th1 cytokine response. The cellular source of IL-4 is the NKT2 subset of invariant NKT (*i*NKT) cells. Consequently, NKT2 deficiency results in the lack of innate CD8 T cells. Whether NKT2 is the only *i*NKT subset and whether IL-4 is the only cytokine required for innate CD8 T cell generation, however, remains unclear. Here, we employed a mouse model of NKT1 deficiency, which is achieved by overexpression of the cytokine receptor IL-2Rβ, and assessed the role of other *i*NKT subsets and cytokines in innate CD8 T cell differentiation. Because IL-2Rβ-transgenic mice failed to generate both NKT1 and innate CD8 T cells, we postulated an *in vivo* requirement for IFNγ-producing NKT1 cells for innate CD8 T cell development. In-depth analyses of IL-2Rβ-transgenic mice and IFNγ-deficient mice, however, demonstrated that neither NKT1 nor IFNγ was required to induce Eomes or to drive innate CD8 T cell generation. Instead, *in vivo* administration of recombinant IL-4 sufficed to restore the development of innate CD8 T cells in NKT1-deficient mice, affirming that intrathymic IL-4, and not IFNγ, is the limiting factor and key regulator of innate CD8 T cell generation in the thymus.

## 1. Introduction

The thymus generates a diverse and self-tolerant repertoire of T cell receptor specificities that is critical for establishing an immunocompetent T cell pool. Newly generated T cells are antigen-inexperienced and functionally immature, so most CD8 T cells require export into peripheral tissues to acquire functional competence and develop effector functions. Nonetheless, some thymic CD8 T cells can obtain innate-like effector functions during their development in the thymus, and they are commonly referred to as innate CD8 T cells [1]. Such innate CD8 T cells are phenotypically distinct from conventional naïve CD8 T cells in the thymus, as they express high levels of the cytokine receptor IL-4Rα and the chemokine receptor CXCR3 but have downregulated the maturation-associated marker CD24 [2,3]. Functionally, innate CD8 T cells produce copious amounts of IFNγ, and they are associated with the expression of large amounts of the transcription factor, eomesodermin (Eomes) [1,4]. Physiologically, innate CD8 T cells are proposed to provide an immediate early immune response to foreign pathogens and to produce proinflammatory cytokines that boost anti-viral immunity [5,6,7,8].

The precise molecular pathway of innate CD8 T cell generation is still disputed, but it has become evident that the availability of intrathymic IL-4 plays a critical role in this process [9,10,11]. The major source of IL-4 has been mapped to the NKT2 subset of *i*NKT cells [11], and the IL-4 requirement for thymic innate CD8 T cell generation is further illustrated by the lack of innate CD8 T cells in mice that are impaired in IL-4 production [5,12]. IL-4 is a prominent member of the common γ-chain (γc) cytokine family and shares multiple downstream signaling pathways with other cytokines of the γc family, such as IL-7 [13]. However, why IL-4 is uniquely required for the generation of innate CD8 T cells and why other cytokines of the γc family fail to replace the IL-4 requirement in innate CD8 T cell differentiation is not known. It also remains unclear if other cytokines, in addition to IL-4, would be necessary for innate CD8 T cell generation. Specifically, a role for IFNγ, the effector cytokine that is produced by both *i*NKT cells and innate CD8 T cells, has not been tested. In this regard, here, we found that innate CD8 T cells express substantially greater amounts of the IFNγ receptor, IFNGR1, than conventional CD8 T cells, which was associated with markedly increased sensitivity to IFNγ and enhanced phosphorylation of STAT1 downstream of IFNγ [14]. Interestingly, we further noted that the lack of the IFNγ-producing *i*NKT cells, i.e., NKT1 cells, correlated with an impaired generation of innate CD8 T cells. However, a causal relationship between the lack of NKT1 and the failure to generate innate CD8 T cells had not been tested.

Thus, we probed in this study a hypothetical model of innate CD8 T cell development in which the initial IFNγ signal would be triggered by NKT1 cells and potentially sustained through an autocrine loop of IFNγ production and signaling by innate CD8 T cells. In-depth analyses of IL-2Rβ-transgenic mice that lack NKT1 cells and IFNγ-deficient mice, however, demonstrated that neither NKT1 cells nor IFNγ production was required for innate CD8 T cell generation in the thymus. Instead, our results demonstrated that the intrathymic availability of IL-4, which is controlled by NKT2 cells, is highly limited, further affirming that IL-4, and not IFNγ, is the critical regulator of innate-like features in developing CD8 T cells.

## 2. Materials and Methods

### 2.1. Mice

BALB/cAnNCrl (BALB/c) mice were obtained from Charles River Laboratories (Frederick, MD, USA). BALB/c *Il4*^−/−^ mice were obtained from Jackson Laboratories (JAX#005879). IL-2Rβ^Tg^ on BALB/c background mice was previously described [15]. *Ifng*^−/−^ mice on a C57BL/6 background have been previously reported [16]; they were procured from the Jackson Laboratories (JAX#002287) and extensively backcrossed to the BALB/c background in this study. All mice were cared for in accordance with NIH guidelines. All animal procedures reported in this study that were performed by NCI-CCR affiliated staff were approved by the NCI Animal Care and Use Committee (ACUC) and in accordance with federal regulatory requirements and standards. All components of the intramural NIH ACU program are accredited by AAALAC International.

### 2.2. Antibodies

Fluorescence-conjugated antibodies with the following specificities were used to detect antigens by flow cytometry: CD4 (GK1.5), CD8α (53-6-7), IL-4Rα (M1), CD44 (IM7), γδT cell receptor (GL3), γc (4G3), RORγt (Q31-378), Runx3 (R3-5G4), pSTAT1 (pY701; 4a), and isotype control antibodies, all from BD Biosciences; CD24 (M1/69), IL-2Rβ (TM-β1), IL-7Rα (A7R34), IFNGR1 (2E2), Eomes (Dan11mag), IL-4 (11B11), IL-17 (eBio17B7) and T-bet (4B10) from Invitrogen; CXCR3 (CXCR3-173), TCRβ (H57-597), IFNγ (XMG1.2), Ikaros (2A9), and PLZF (9E12) from Biolegend. CD1d tetramers loaded with PBS-57 were obtained from the NIH tetramer facility (Emory University, Atlanta, GA, USA).

### 2.3. Cell Isolation

Thymocytes were isolated by teasing apart thymuses with tweezers and then resuspending the processed cell suspension in harvest media (10% FCS in RPMI-1640). Thymocyte suspensions were filtered through 100 µm Nylon filter meshes (Millipore Sigma, Burlington, MA, USA) to remove tissue debris. Cell filtrates were washed once in harvest media by centrifugation for 7 min at 1500 rpm, and the pellet was resuspended in FACS buffer (0.5% BSA, 0.1% sodium azide in HBSS) before staining.

### 2.4. Flow Cytometry

Single-cell suspensions were stained with fluorescence-conjugated antibodies and analyzed by flow cytometry as previously described [17]. In brief, 3–5 × 10^6^ thymocytes were washed with FACS buffer, and the cells were then stained with the indicated antibodies for 30 min at 4 °C. For *i*NKT cell staining, we first performed CD1d tetramer staining for 20 min at 4 °C and then added other antibodies for surface protein detection. Thereafter, the cells were washed twice with FACS buffer before analysis by flow cytometry. Samples were analyzed using LSRII or LSR Fortessa flow cytometers (BD Bioscience, Franklin Lakes, NJ, USA). Flow cytometry data were analyzed using the FlowJo v10.6.2 software (FlowJo, LLC, Ashland, OR, USA) using gating strategies, as laid out in Figure S1.

### 2.5. Intracellular Cytokine Expression Assays

Intracellular staining to assess cytokine production was performed as previously described [18]. In brief, thymocytes were resuspended into a concentration of 5 × 10^6^ cells/mL in 10% FCS-supplemented cell culture media in 24-well flat-bottom plates and stimulated with PMA (50 µg/mL) and ionomycin (1 µM) for 4 h in the presence of Brefeldin A (BFA; 3 µg/mL) in a 7.5% CO_2_ atmosphere in a 37 °C incubator. Stimulation was stopped by washing the cells with cold PBS. Afterwards, cells were counterstained with viability dye (Ghost Dye Violet 510). After 20 min, the cells were washed with FACS buffer. Surface proteins were stained with the indicated antibodies for 30 min at 4 °C, and cells were subsequently washed and fixed with IC fixation buffer (Invitrogen, Carlsbad, CA, USA) for 20 min at room temperature. Next, the cells were washed twice with permeabilization buffer (Invitrogen), and cytokine staining was conducted for 1 h at room temperature. Finally, the cells were washed with FACS buffer before their flow cytometric analysis.

### 2.6. Nuclear Transcription Factor Staining

*i*NKT cells were stained with PBS-57-loaded CD1d tetramers for 20 min at 4 °C, after which other antibodies for surface proteins were added. After 30 min, the stained cells were washed once with FACS buffer and fixed with 100 µL FACS buffer and 150 µL of a 1:3 mixture of a concentrate/diluent solution from the Foxp3/Transcription Factor Staining Buffer kit (eBioscience Thermo Fisher, San Diego, CA, USA) for 20 min at room temperature. The cells were washed again twice with permeabilization buffer before incubation with antibodies against nuclear transcription factors, such as PLZF, T-bet, and RORγt. After 60 min, the cells were washed again and analyzed using flow cytometry.

### 2.7. In-Vivo Treatment of IL-2Rβ^Tg^ Mice with IL-4 and Anti-IL-4 Antibody Complex

IL-2Rβ^Tg^ mice were *i.p.* injected with 1 µg of recombinant IL-4 (Peprotech, Cranbury, NJ, USA) complexed with anti-IL-4 antibodies (eBioscience) or vehicle every other day for 2 weeks. An IL-4 and anti-IL-4 antibody complex was prepared as previously described [19] with slight modifications. Then, 20 µg of recombinant IL-4 (Peprotech) powder was reconstituted with 80 µL distilled water and mixed with 120 µL anti-IL-4 antibody (1 mg/mL; 11B11; eBioscience). The mixture was incubated for 10 min at room temperature, diluted 10-fold with PBS, and kept at 4 °C until further use.

### 2.8. Co-Staining for Intracellular pSTAT1/Eomes

The codetection of pSTAT1 and nuclear Eomes was performed using a protocol that we previously reported [20]. In brief, thymocytes were resuspended to a concentration of 5 × 10^6^ cells/mL in PBS and counterstained with viability dye (Ghost Dye Violet 510) for 20 min. Cells were then stimulated with different amounts of recombinant IFNγ (0.1, 0.3, and 1 ng/mL) and incubated for 30 min at 37 °C in serum-free RPMI-1640 media. After washing the cells with permeabilization buffer (Invitrogen), intracellular staining for Eomes was conducted for 30 min at room temperature. Next, cells were fixed and permeabilized with cold 4% PFA in PBS followed by ice-cold 90% methanol for 30 min on ice. Afterward, cells were washed twice with FACS buffer and restained with the isotype control and pSTAT1 antibody for 1 h at room temperature. Next, surface antibodies were added, and cells were incubated for an additional 20 min. Finally, the stained cells were washed with FACS buffer and filtered before flow cytometric analysis.

### 2.9. Statistics

Statistical analyses were performed using Prism 9.3.1 software (GraphPad, San Diego, CA, USA). Comparisons between groups were analyzed using the Student’s *t*-test or Mann–Whitney test. Data are presented as the mean ± SEM. *p* values of less than 0.05 were considered significant (* *p* < 0.05, ** *p* < 0.005, *** *p* < 0.0005).

## 3. Results

### 3.1. Distinct Cytokine Requirements for IL-4-Producing iNKT and IFNγ-Expression Innate CD8 T Cells

**Figure 1:**
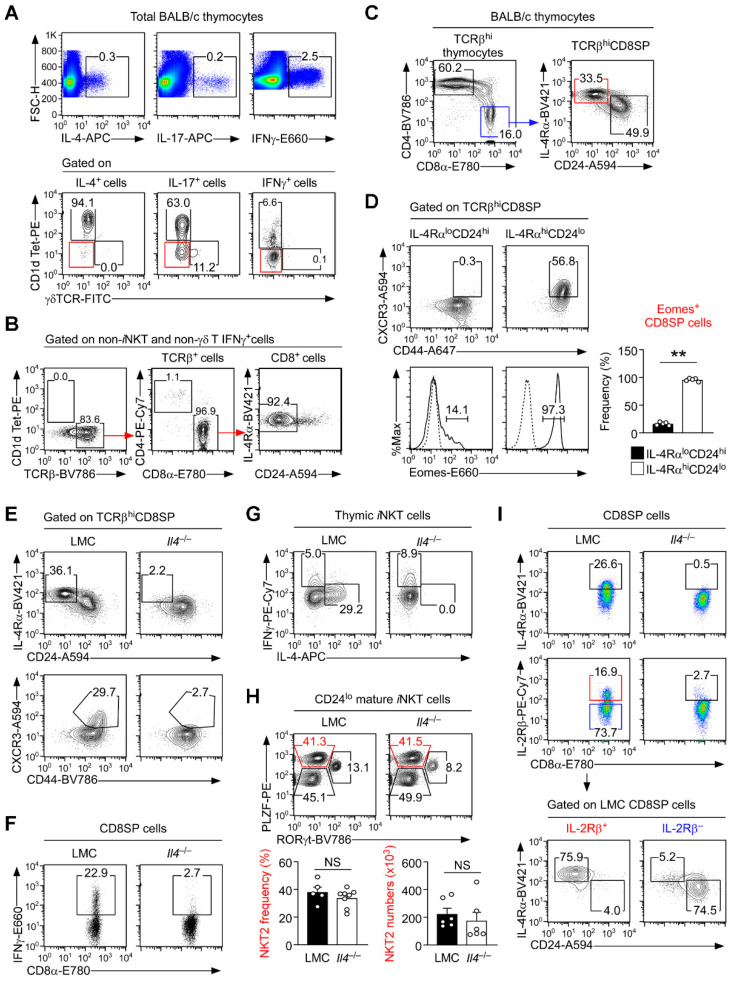
Innate CD8 T cells are the main producers of intrathymic IFNγ. (**A**) The identities of IL-4- (**left**), IL-17- (**middle**), and IFNγ-producing cells (**right**) were assessed in PMA + ionomycin stimulated BALB/c thymocytes by assessing CD1d-tetramer and γδ TCR staining (bottom). Numbers in boxes indicate the relative frequency of *i*NKT cells (CD1dTet^+^) and γδ T cells (γδTCR). The results are representative of four independent experiments. (**B**) IFNγ-producing non-*i*NKT, non-γδ T cells were further analyzed for their cellular identity, revealing them as αβ T cells (**left**) that are exclusively CD8 lineage cells (**middle**), expressing an IL-4Rα^hi^CD24^lo^ innate-like phenotype (**right**). The results are representative of two independent experiments. (**C**) Innate and conventional CD8 T cells (**right**) are identified by their distinct IL-4Rα and CD24 expression (**right**) among TCRβ^hi^ CD8SP thymocytes (**left**) of BALB/c mice. Results are representative of 10 independent experiments. (**D**) Intracellular Eomes expression in innate (IL-4Rα^hi^CD24^lo^) and naïve (IL-4Rα^lo^CD24^hi^) CD8 T cells among BALB/c thymocytes. The results are representative of three independent experiments. (**E**) CD24 versus IL-4Rα expression and CD44 versus CXCR3 expression were assessed on TCRβ^hi^ CD8SP thymocytes of *Il4*^−/−^ and LMC BALB/c mice. Numbers in gates represent the frequencies of innate CD8 T cells. The results are representative of four independent experiments with a total of seven *Il4*^−/−^ and five LMC BALB/c mice. (**F**) IFNγ expression was assessed in PMA- and ionomycin-stimulated CD8SP thymocytes of *Il4*^−/−^ and LMC BALB/c mice. The results are representative of three independent experiments with a total of six *Il4*^−/−^ and five LMC BALB/c mice. (**G**) IFNγ and IL-4 production was assessed in PMA- and ionomycin-stimulated thymic *i*NKT cells of *Il4*^−/−^ and LMC BALB/c mice. The results are representative of three independent experiments with a total of six *Il4*^−/−^ and five LMC BALB/c mice. (**H**) Thymic *i*NKT subset compositions were assessed in CD24^lo^ mature *i*NKT cells of *Il4*^−/−^ and LMC BALB/c mice by RORγt versus PLZF staining. Contour plots are representative (**top**), and bar graphs of NKT2 cell frequencies and numbers (**bottom**) show a summary of four independent experiments with a total of at least six *Il4*^−/−^ and five LMC BALB/c mice. (**I**) Expression of the cytokine receptors IL-4Rα and IL-2Rβ on mature CD8SP thymocytes of *Il4*^−/−^ and LMC BALB/c mice (**top**). IL-2Rβ-positive (IL-2Rβ^+^, red box) and IL-2Rβ-negative (IL-2Rβ^−^, blue box) CD8SP cells of LMC mice were further assessed for CD24 versus IL-4Rα expression (**bottom**). The results are representative of eight independent experiments. Numbers in contour plots and dot plots indicate the frequencies of cells within the corresponding boxes. Statistical significance in (**D**,**H**); Mann–Whitney test, (** *p* < 0.005).

The intrathymic sources of immunoregulatory cytokines dramatically differ depending on their identities [21]. In BALB/c mice, we found that IL-4 is almost exclusively expressed by *i*NKT cells, and we identified *i*NKT cells as a major source of intrathymic IL-17 as well (Figure 1A) [22]. On the other hand, in the same BALB/c mice, IFNγ is principally produced by αβ T lineage cells that are not *i*NKT cells (Figure 1A and Figure S2). Further analyses identified these intrathymic IFNγ producers as CD8 T cells that express high levels of IL-4Rα but low amounts of CD24 (Figure 1B), which are commonly referred to as thymic innate CD8 T cells [8,23,24]. Innate CD8 T cells are distinct from naïve CD8 T cells, which express low-levels of IL-4Rα but large amounts of CD24 (Figure 1C). Innate CD8 T cells (IL-4Rα^hi^CD24^lo^) differ from naïve CD8 T cells (IL-4Rα^lo^CD24^hi^) also by their high-level expression of CXCR3, CD44, and Eomes (Figure 1D) [24,25]. Innate CD8 T cells depend on intrathymic IL-4 for their development, as illustrated in IL-4-deficient mice that lack IL-4Rα^hi^CD24^lo^ and CXCR3^hi^CD44^hi^ innate-phenotype (Figure 1E) and IFNγ-producing CD8 T cells (Figure 1F and Figure S3A,B). Moreover, it is the failure to produce IL-4 and not the lack of NKT2 cells that is responsible for the paucity of innate CD8 T cells because *i*NKT cells in *Il4*^−/−^ mice do not produce IL-4 (Figure 1G), but the frequency and number of PLZF^hi^ NKT2 cells remain unchanged between *Il4*^−/−^ and littermate control (LMC) mice (Figure 1H and Figure S2C). Thus, IL-4 plays a critical role in innate CD8 T cell generation but is dispensable for NKT2 cell differentiation.

At this point, it is unclear why specifically IL-4 is necessary for innate CD8 T cell generation. A major effect of IL-4 signaling is the upregulation of IL-4 receptor expression [26], which coincides with the large abundance of IL-4Rα on innate CD8 T cells (Figure 1I) [2,11]. To examine whether γc family cytokine receptors other than IL-4Rα are also subject to IL-4-induced upregulation, we assessed the expression of IL-2Rβ, IL-7Rα, and γc on *Il4*^−/−^ and LMC CD8 single positive (CD8SP) thymocytes (Figure 1I and Figure S3D). Importantly, while IL-2Rβ^+^ cells mostly corresponded to innate CD8 T cells, IL-2Rβ^−^ cells were mostly comprised of naïve CD8 T cells (Figure 1I, bottom). Thus, we postulated that IL-4-mediated innate CD8 T cell differentiation could be associated with IL-2Rβ expression. IL-2Rβ is a shared receptor subunit for IL-2 and IL-15, whose abundance directly affects cellular responsiveness to these cytokines [27]. Both IL-2 and IL-15 play critical roles in driving CD8 effector/memory cell differentiation [28,29]. Accordingly, we next questioned whether IL-4 could be necessary for innate CD8 T cell generation because it upregulates the expression of IL-2Rβ, potentiating their responsiveness to IL-2 and IL-15. If so, we further aimed to test whether the forced overexpression of IL-2Rβ would be sufficient to induce innate CD8 T cell differentiation.

### 3.2. Forced Expression of IL-2Rβ Suppresses the Generation of Innate CD8 T Cells

**Figure 2:**
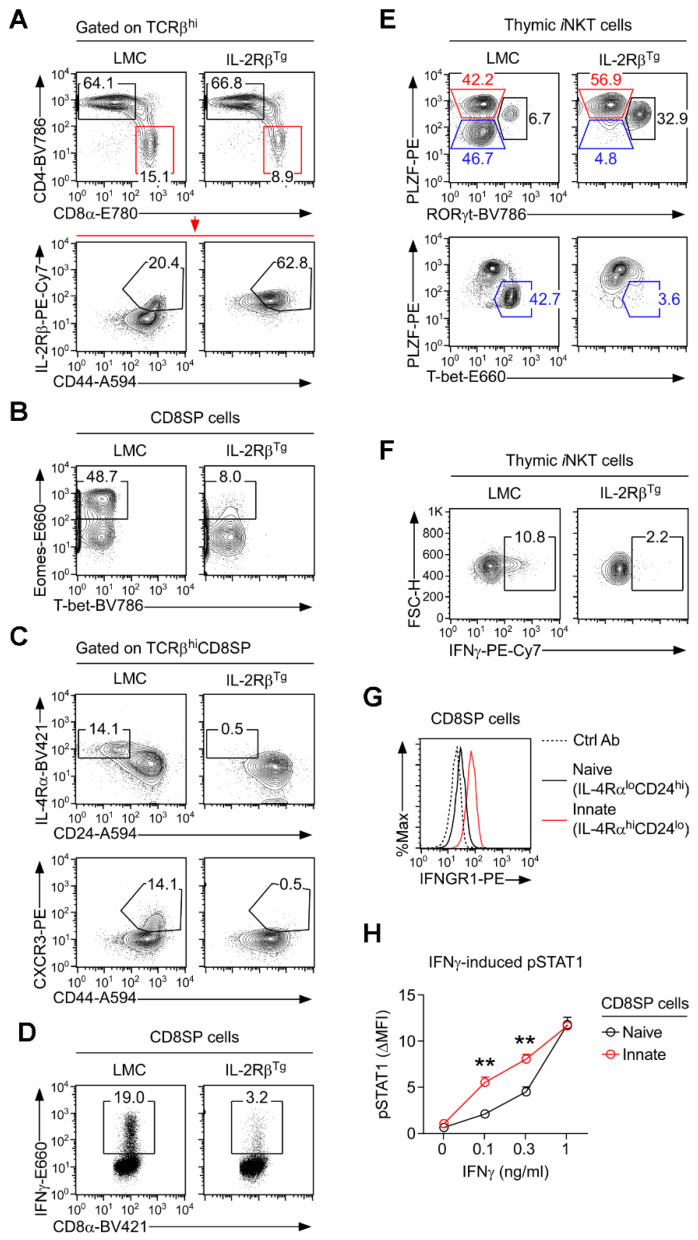
The forced expression of IL-2Rβ inhibits the generation of innate CD8 T cells. (**A**) Mature CD8SP cells were identified among TCRβ^hi^ thymocytes of IL-2Rβ^Tg^ and LMC BALB/c mice (**top**) and assessed for IL-2Rβ versus CD44 expression (**bottom**). The results are representative of 6 independent experiments with a total of 11 IL-2Rβ^Tg^ and 12 LMC BALB/c mice. (**B**) Nuclear staining for Eomes and T-bet in CD8SP thymocytes of IL-2Rβ^Tg^ and LMC BALB/c mice. The results are representative of three independent experiments with a total of five IL-2Rβ^Tg^ and five LMC BALB/c mice. (**C**) CD24 versus IL-4Rα and CD44 versus CXCR3 expression were assessed on TCRβ^hi^ CD8SP thymocytes of IL-2Rβ^Tg^ and LMC BALB/c mice. The numbers in gates represent the frequencies of innate CD8 T cells. The results are representative of 5 independent experiments with a total of 10 IL-2Rβ^Tg^ and 11 LMC BALB/c mice. (**D**) IFNγ expression was assessed in PMA- and ionomycin-stimulated CD8SP thymocytes of IL-2Rβ^Tg^ and LMC BALB/c mice. Dot plots are representative of three independent experiments with a total of six IL-2Rβ^Tg^ and six LMC BALB/c mice. (**E**) The subset compositions of thymic *i*NKT cells in IL-2Rβ^Tg^ and LMC BALB/c mice were assessed by RORγt versus PLZF staining (**top**) and T-bet versus PLZF staining (**bottom**). The results are representative of four independent experiments with a total of nine IL-2Rβ^Tg^ and ten LMC BALB/c mice. (**F**) IFNγ production was assessed in PMA- and ionomycin-stimulated thymic *i*NKT cells of IL-2Rβ^Tg^ and LMC BALB/c mice. The results are representative of three independent experiments with a total of six IL-2Rβ^Tg^ and six LMC BALB/c mice. (**G**) IFNγ receptor (IFNGR1) expression was quantified on innate (IL-4Rα^hi^CD24^lo^) and naïve (IL-4Rα^lo^CD24^hi^) CD8 T cells of BALB/c thymocytes. Histograms are representative of 2 independent experiments. (**H**) STAT1 phosphorylation (pSTAT1) in naïve (Eomes^−^) versus innate (Eomes^+^) CD8 T cells of BALB/c thymocytes upon 30 min in-vitro stimulation with increased amounts of recombinant IFNγ (0.1, 0.3, and 1 ng/mL). The graph shows the summary of two independent experiments with a total of four BALB/c mice. Numbers in contour plots and dot plots indicate the frequencies of cells within the corresponding boxes. Statistical significance in (**H**); Mann–Whitney test, (** *p* < 0.005).

To address these questions, we analyzed CD8 T cell development in IL-2Rβ^Tg^ mice, in which IL-2Rβ is overexpressed in all thymocytes, including mature CD8SP cells (Figure 2A) [15]. Surprisingly, and contrary to our expectation, we found that the generation of CD8 T cells in general (Figure S4A), and of innate CD8 T cells specifically, was not increased in BALB/c mice overexpressing the IL-2Rβ. Instead, innate CD8 T cell differentiation was virtually abolished, as evidenced by the loss of Eomes^+^ CD8SP cells (Figure 2B), the lack of IL-4Rα^hi^CD24^lo^ and CXCR3^hi^CD44^hi^ CD8SP thymocytes (Figure 2C), and the inability of CD8 T cells to produce IFNγ (Figure 2D and Figure S4B,C). The overexpression of IL-2Rβ in C57BL/6 mice, on the other hand, did not alter innate CD8 T cell generation because CD57BL/6 mice are mostly devoid of innate CD8 T cells (Figure S3D,E). Collectively, the forced expression of IL-2Rβ did not promote, but paradoxically suppressed, the generation of innate CD8 T cells.

### 3.3. Lack of Innate CD8 T Cells in IL-2Rβ^Tg^ Mice Is Associated with the Lack of IFNγ Expression

To understand why innate CD8 T cell differentiation is impaired in IL-2Rβ^Tg^ mice, we next considered the possibility that the forced expression of IL-2Rβ would interfere with the generation of NKT2 cells. In IL-2Rβ^Tg^ BALB/c mice, the overall thymic *i*NKT subset composition was indeed dysregulated (Figure 2E, top) [15]. While NKT2 cell differentiation appeared to be intact (Figure 2E, top), the generation of NKT1 cell was dramatically impaired (Figure 2E, bottom). Altogether, IL-2Rβ^Tg^ thymocytes not only lacked IFNγ-producing innate CD8 T cells but also IFNγ-producing NKT1 subset *i*NKT cells (Figure 2F), resulting in the dramatic loss of overall intrathymic IFNγ production (Figure S4C). These findings prompted us to consider the possibility that intrathymic IFNγ could be required for and would contribute to innate CD8 T cell differentiation. To this end, we quantified the expression of IFNGR1, i.e., the IFNγ receptor [30], on naïve versus innate CD8 T cells. Here, we found that IFNGR1 was highly abundant on innate CD8 T cells (Figure 2G and Figure S4F). Accordingly, IFNγ responsiveness was substantially increased in innate CD8 T cells compared to conventional CD8 T cells, as shown by the increased STAT1 phosphorylation downstream of in-vitro IFNγ signaling (Figure 2H). Altogether, these results reveal a previously unappreciated aspect in IFNγ sensitivity that is significantly increased in innate CD8 T cells compared to naïve CD8 T cells.

### 3.4. IFNγ Is Dispensable for the Generation of Innate CD8 T Cells in the Thymus

**Figure 3:**
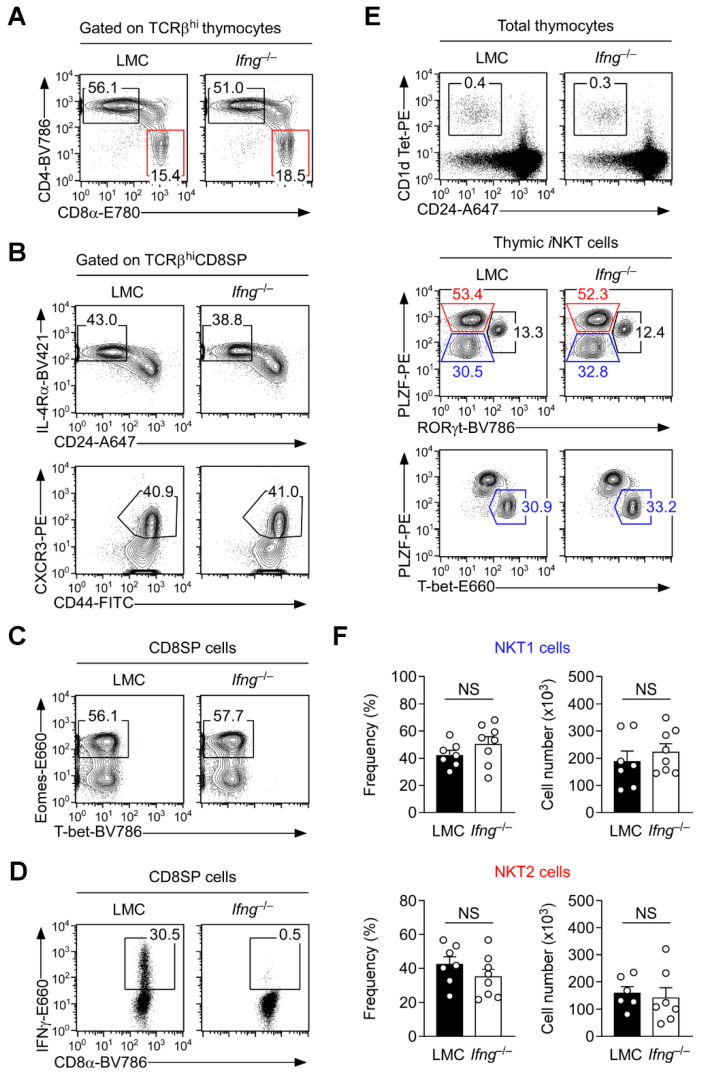
IFNγ is dispensable for the generation of both NKT1 and innate CD8 T cells. (**A**) The frequencies of mature CD4SP and CD8SP cells were assessed among TCRβ^hi^ thymocytes of *Ifng*^−/−^ and LMC BALB/c mice. The results are representative of four independent experiments with a total of ten *Ifng*^−/−^ and seven LMC BALB/c mice. (**B**) CD24 versus IL-4Rα (**top**) and CD44 versus CXCR3 expression (**bottom**) were examined in TCRβ^hi^ CD8SP thymocytes of *Ifng*^−/−^ and LMC BALB/c mice. Numbers in gates represent the frequencies of innate CD8 T cells. The results are representative of three independent experiments with a total of seven *Ifng*^−/−^ and seven LMC BALB/c mice. (**C**) Nuclear staining for Eomes and T-bet in CD8SP thymocytes of *Ifng*^−/−^ and LMC BALB/c mice. The results are representative of three independent experiments. (**D**) IFNγ expression was assessed in PMA- and ionomycin-stimulated CD8SP thymocytes of *Ifng*^−/−^ and LMC BALB/c mice. The results are representative of three independent experiments. (**E**) Thymic *i*NKT subset compositions were assessed in CD24^lo^ *i*NKT cells (**top**) *Ifng*^−/−^ and LMC BALB/c mice by intracellular RORγt versus PLZF (**middle**) and T-bet versus PLZF staining (**bottom**). The results are representative of three independent experiments with a total of six *Ifng*^−/−^ and six LMC BALB/c mice. (**F**) Frequencies and numbers of NKT1 (**top**) and NKT2 cells (**bottom**) *Ifng*^−/−^ and LMC BALB/c mice. The results are representative of three independent experiments with a total of at least seven *Ifng*^−/−^ and eight LMC BALB/c mice. Numbers in contour plots and dot plots indicate the frequencies of cells within the corresponding boxes. Statistical significance in Figure 3F; Mann–Whitney test.

To directly test a causal relationship between IFNγ and innate CD8 T cells, we next analyzed thymocyte development in IFNγ-deficient (*Ifng*^−/−^) BALB/c mice. Contrary to our expectations, the generation of mature CD8SP cells (Figure 3A and Figure S5A) and the differentiation of innate CD8 T cells were not diminished in *Ifng*^−/−^ BALB/c mice, as demonstrated by the robust presence of IL-4Rα^hi^CD24^lo^ and CXCR3^hi^CD44^hi^ CD8SP thymocytes (Figure 3B and Figure S5B) and Eomes^+^ CD8SP cells (Figure 3C and Figure S5C). While they phenotypically corresponded to innate CD8 T cells, *Ifng*^−/−^ CD8 T cells were functionally impaired because they failed to produce IFNγ due to their genetic deficiency in *Ifng* (Figure 3D and Figure S5D). Moreover, thymic *i*NKT cell development and the *i*NKT subset composition of *Ifng*^−/−^ and LMC mice did not differ between each other (Figure 3E and Figure S5E), so that both the frequency and number of NKT1 and NKT2 cells were unaffected by IFNγ deficiency (Figure 3F). Collectively, these results show that the thymic generation of NKT1 cells as well as innate CD8 T cells does not require IFNγ. Moreover, the innate CD8 T cells generated in *Ifng*^−/−^ and LMC BALB/c mice did not differ in their surface molecule phenotype or major transcription factor expression either (Figure S5F). Thus, the subset-specific cytokine expression by *i*NKT cells is relevant in inflammation (Figure S6), but the IFNγ production of thymic NKT 1 cells is dispensable for thymic innate CD8 T cells. These results indicate that increased IFNγ sensitivity is a consequence rather than the cause of innate CD8 T cell differentiation.

### 3.5. Innate CD8 T Cell Development Is Controlled by the Abundance of Intrathymic IL-4

**Figure 4:**
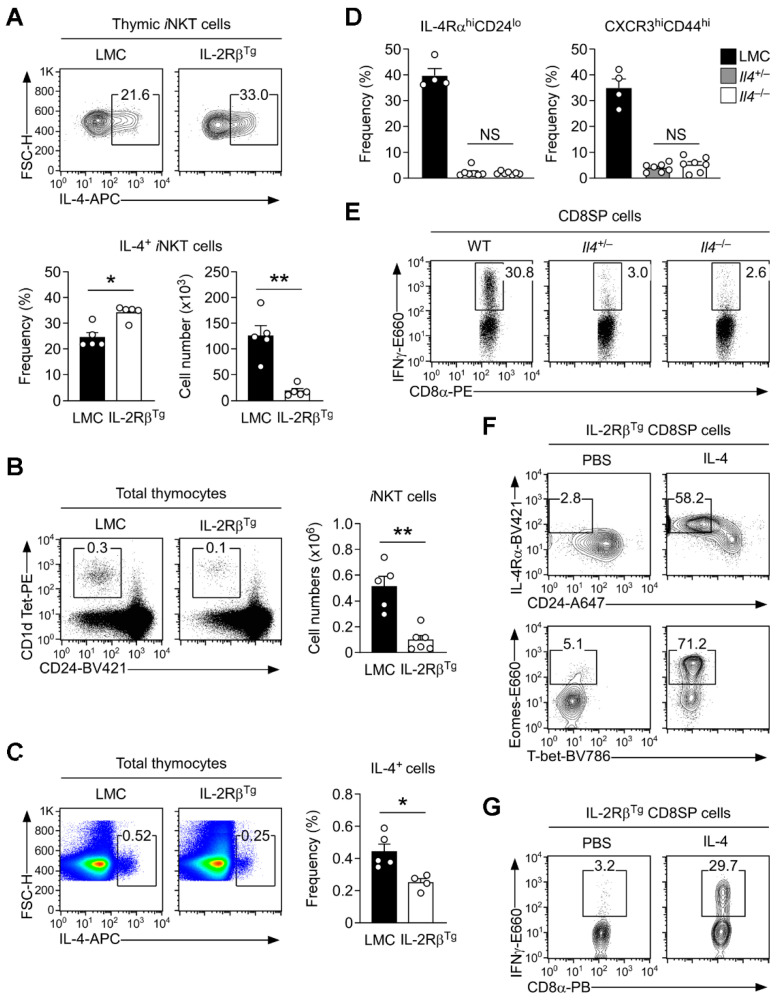
The abundance of intrathymic IL-4 controls innate CD8 T cell generation. (**A**) Frequencies and numbers of IL-4-producing *i*NKT cells in IL-2Rβ^Tg^ and LMC BALB/c mice. Contour plots are representative (**top**) and bar graphs (**bottom**) are the summary of three independent experiments with a total of five IL-2Rβ^Tg^ and five LMC BALB/c mice. (**B**) Mature thymic *i*NKT cells in IL-2Rβ^Tg^ and LMC BALB/c mice. The dot plots are representative (**left**) and the bar graph (**right**) is the summary of five independent experiments with a total of six IL-2Rβ^Tg^ and five LMC BALB/c mice. (**C**) Frequencies of IL-4-producing cells in IL-2Rβ^Tg^ and LMC BALB/c thymocytes. The dot plots are representative (**left**) and the bar graph (**right**) is the summary of three independent experiments with a total of four IL-2Rβ^Tg^ and five LMC BALB/c mice. (**D**) Frequencies of IL-4Rα^hi^CD24^lo^ and CXCR3^hi^CD44^hi^ innate CD8 T cells in *Il4*^−/−^, *Il4*^+/−^ and LMC BALB/c CD8SP thymocytes. Graphs represent the summary of three independent experiments with a total of seven *Il4*^−/−^, seven *Il4*^+/−^ and four LMC BALB/c mice. (**E**) IFNγ expression in PMA- and ionomycin-stimulated CD8SP thymocytes of *Il4*^−/−^, *Il4*^+/−^, and WT BALB/c mice. Dot plots are representative of four independent experiments with ten *Il4*^−/−^, six *Il4*^+/−^, and four WT BALB/c mice. (**F**) Cell surface expression of CD24 versus IL-4Rα (**top**) and intracellular expression of Eomes versus T-bet were assessed in mature CD8SP thymocytes of IL-2Rβ^Tg^ mice after recombinant IL-4 or vehicle control (PBS) injection. Results are representative of two independent experiments with a total of three IL-2Rβ^Tg^ mice. (**G**) IFNγ expression in PMA- and ionomycin-stimulated CD8SP thymocytes of IL-2Rβ^Tg^ mice after recombinant IL-4 or vehicle control (PBS) injection. The results are representative of two independent experiments with a total of three IL-2Rβ^Tg^ mice. Numbers in contour plots and dot plots indicate the frequencies of cells within the corresponding boxes. Statistical significance in Figure 4A; Student’s *t*-test. Statistical significance in (**B**–**D**); Mann–Whitney test, (* *p* < 0.05, ** *p* < 0.005).

To explain the lack of innate CD8 T cells in IL-2Rβ^Tg^ mice, we next examined cytokine production in IL-2Rβ^Tg^ *i*NKT cells. While IL-4 production itself was intact (Figure 4A, top), notably, the number of IL-4-producing *i*NKT cells was significantly decreased compared to that of LMC mice (Figure 4A, bottom right). The reason for the decrease is related to the diminished number of *i*NKT cells in IL-2Rβ^Tg^ mice (Figure 4B). In fact, the overall number of CD24^lo^ mature *i*NKT cells was significantly decreased in IL-2Rβ^Tg^ mice (Figure 4B), and because intrathymic IL-4 is almost exclusively produced by *i*NKT cells (Figure 1A), there was a substantial decrease in total IL-4-producing thymocytes (Figure 4C). These results suggested that the abundance of intrathymic IL-4 is scarce and that its availability constrains the generation of innate CD8 T cells.

If such were the case, we next asked whether diminishing IL-4’s availability would further constrain the generation of innate CD8 T cells. To address this question, we assessed the frequency of innate CD8 T cells in IL-4 heterozygote (*Il4*^+/−^) mice. *Il4*^+/−^ BALB/c mice are still capable of producing IL-4, albeit at reduced levels, but strikingly, these mice were profoundly impaired in innate CD8 T cell generation to the same degree as observed in complete IL-4-deficient (*Il4*^−/−^) mice (Figure 4D and Figure S7A). Accordingly, *Il4*^+/−^ mice also lacked IFNγ-producing CD8SP thymocytes, as is the case for Il4^−/−^ mice (Figure 4E and Figure S7B). Collectively, these results indicate that innate CD8 T cell development is highly sensitive and strictly dependent on the abundance of intrathymic IL-4.

To directly demonstrate that IL-4 determines the size of the innate CD8 T cell pool, we next administrated recombinant IL-4 proteins to IL-2Rβ^Tg^ BALB/c mice, asking whether an increase in IL-4 availability would restore innate CD8 T cell generation. Strikingly, this was precisely what we observed after injecting bioactive IL-4 proteins into these mice. We found a dramatic increase in IL-4Rα^hi^CD24^lo^ and Eomes^+^ innate-like CD8 T cells (Figure 4F) without significant changes in the thymic *i*NKT subset composition (Figure S8). Importantly, such phenotypically innate CD8 T cells were also functionally competent as they produced large amounts of IFNγ upon their activation (Figure 4G). These results reveal that IFNγ is dispensable, but IL-4 is critically required for the generation of innate CD8 T cells. Our findings further divulge intrathymic IL-4 as a scarce commodity whose abundance directly controls the number of innate CD8 T cells in the thymus.

## 4. Discussion

Innate CD8 T cells correspond to memory-phenotype (MP) CD8 T cells, whose generation is considered mostly cytokine-driven and not an antigen-driven event [31]. Depending on the anatomical location where these cells arise and the identity of the cytokine that drives their differentiation, several distinct populations of MP CD8 T cells have been proposed [32,33]. Accordingly, “virtual memory” T cells are generated in peripheral tissues by IL-15 signaling, whereas “homeostatic memory” T cells are produced via lymphopenia-induced proliferation in peripheral organs in an IL-7- and IL-15-dependent manner [1,34]. In contrast to MP CD8 T cells in peripheral tissues, innate CD8 T cells are commonly referred to an MP population of T cells that develops and resides in the thymus and that uniquely depends on IL-4 for their generation [1,3]. Why IL-4 is specifically required for thymic innate CD8 T cells, and whether IL-4 is the only cytokine that is required for their generation, are some of the issues that have not been fully resolved.

To address these questions, we considered it important to map the downstream targets of IL-4 signaling in CD8 thymocytes to identify molecules that are associated with innate CD8 T cell differentiation. While the transcriptional landscape of naïve versus innate CD8 T cells has been previously reported [35], here, we specifically focused on distinct cytokine receptor expression between IL-4-dependent innate CD8 T cells and IL-4-independent conventional CD8 T cells. Notably, we found that the expression of the cytokine receptors IL-2Rβ, IL-4Rα, and IFNGR1 were highly induced on innate CD8 T cells compared to naïve CD8 T cells. Among these, we were intrigued by the increased surface abundance of the IFNγ receptor. Innate CD8 T cells produce copious amounts of IFNγ, which is the ligand of the IFNγ receptor, whereas they do not produce IL-2/IL-15 and IL-4, which are the ligands of IL-2Rβ and IL-4Rα. Therefore, IFNγ is both consumed and produced by innate CD8 T cells, suggesting a possible autocrine circuitry of IFNγ signaling and production in innate CD8 T cells.

Built on these observations, we asked whether such a feed-forward mechanism of IFNγ signaling would indeed exist in innate CD8 T cells, and whether its operation would be necessary for their development [14]. Under this scenario, IFNγ that is produced by non-innate CD8 T cells would first trigger IFNγ signaling in thymocytes that have committed to innate CD8 lineage cells. An IFNγ autocrine pathway would then further induce IFNγ expression and upregulate IFNGR1 expression to drive the end-differentiation into mature innate CD8 T cells. The analyses of IFNγ-deficient BALB/c mice, however, argued against such a scenario and documented this not being the case. Specifically, we found that IFNγ deficiency did not impair the generation of innate CD8 T cells, and that innate CD8 T cells in *Ifng*^−/−^ BALB/c mice were phenotypically indistinguishable to their WT counterparts regarding CD24, CXCR3, IL-4Rα, and CD44 expression as well as Eomes and other nuclear factor expression. Thus, IFNγ is clearly not a developmental requirement for IFNγ-producing innate CD8 T cell generation. In a similar manner, IFNγ is not required for the generation of IFNγ-producing NKT1 cells either.

Analogous to these findings, we further found that IL-4 is also not a developmental requirement for IL-4-produing NKT2 cells. Because NKT2 cells represent the major source of intrathymic IL-4, the number and frequency of NKT2 cells directly control innate CD8 T cell differentiation [11]. Along these lines, it would be critical to understand the molecular mechanism of how NKT2 cell differentiation is regulated in the thymus. There is a clear indication that the genetic backgrounds of different mouse strains affect *i*NKT subset differentiation and, thus, the abundance of NKT2 cells [11,12]. However, other genetic factors and transcriptional control mechanisms also play significant roles in this process. In this regard, it was recently shown that positive selection and subset differentiation of thymic *i*NKT cells are temporally separated events, in which transient TCR signaling is critical for positive selection, while subsequent cytokine signaling is presumably involved in determining the *i*NKT subset identity [36]. Unlike the roles of IL-15 in NKT1 cells and TGFβ in NKT17 cells [37,38], however, the identity of the cytokine(s) that specifies NKT2 cell differentiation is unclear. Because NKT2 cells express large amounts of IL-17RB (the cytokine receptor for IL-25), it was previously proposed that IL-25 could be involved in NKT2 cell differentiation [39,40]. However, IL-17RB is also found on NKT17 cells [39,41], and so it is doubtful that IL-25 signaling selectively drives NKT2 cell generation. Thus, the cytokine requirement of NKT2 cells still needs to be examined. In this regard, our current study showed that at least IL-4 and IFNγ can be excluded as requirements in the cytokine-driven subset specification of thymic NKT2 cells. Further studies will be necessary to fully map this pathway.

Our data also affirmed that innate CD8 T cell generation is highly sensitive to the abundance of intrathymic IL-4. Consequently, previous observations, such as the acute induction of innate CD8 T cells by systemic inflammatory immune responses, can be explained by an increase in the availability of intrathymic IL-4 [7]. Nonetheless, the physiological role of increased thymic innate CD8 T cells in response to increased IL-4 expression remains unclear. Because innate CD8 T cells respond to the Th2 cytokine IL-4 but produce the Th1 cytokine IFNγ, it is feasible that innate CD8 T cells act as rheostats of the intrathymic cytokine milieu. As such, elevated levels of IL-4 would increase the abundance of innate CD8 T cells, which in turn would increase the consumption of IL-4 to neutralize Th2 cytokine effects while triggering an increased Th1 response to balance cytokine expression. The existence of such a regulatory scenario in innate CD8 T cells still needs to be experimentally tested. However, it is evident that the cytokine consumption and production of innate CD8 T cells are unique among thymocytes, and that understanding their roles in thymopoiesis and thymocyte development remains a critical issue in T cell immunology.

## Data Availability

All data and materials will be available upon request.

## Supplementary Materials

The following supporting information can be downloaded at: https://www.mdpi.com/article/10.3390/cells12202433/s1, Figure S1: Representative gating strategies for thymic innate CD8 T cells and *i*NKT subsets. Figure S2: T cell subset distribution of cytokine-producing BALB/c thymocytes. Figure S3: CD8 T cell development in *Il4*^−/−^ BALB/c mice. Figure S4: CD8 T cell development in IL-2Rβ^Tg^ BALB/c mice. Figure S5: Thymic innate CD8 T cell generation in IFNγ-deficient mice. Figure S6: IL-4 and IFNγ expression in NKT1 and NKT2 cells. Figure S7: Innate CD8 T cells and thymic *i*NKT cells of IL-4-deficient mice. Figure S8: Thymic *i*NKT cells of IL-4-treated IL-2Rβ^Tg^ BALB/c mice.

## References

[1] Jameson S.C. (2021). The Naming of Memory T-Cell Subsets. Cold Spring Harb. Perspect. Biol..

[2] Weinreich M.A., Odumade O.A., Jameson S.C., Hogquist K.A. (2010). T cells expressing the transcription factor PLZF regulate the development of memory-like CD8+ T cells. Nat. Immunol..

[3] Park J.Y., Won H.Y., DiPalma D.T., Hong C., Park J.H. (2021). Protein abundance of the cytokine receptor γc controls the thymic generation of innate-like T cells. Cell. Mol. Life Sci..

[4] Jacomet F., Cayssials E., Basbous S., Levescot A., Piccirilli N., Desmier D., Robin A., Barra A., Giraud C., Guilhot F. (2015). Evidence for eomesodermin-expressing innate-like CD8(+) KIR/NKG2A(+) T cells in human adults and cord blood samples. Eur. J. Immunol..

[5] Lee A., Park S.P., Park C.H., Kang B.H., Park S.H., Ha S.J., Jung K.C. (2015). IL-4 Induced Innate CD8+ T Cells Control Persistent Viral Infection. PLoS Pathog..

[6] Renkema K.R., Lee J.Y., Lee Y.J., Hamilton S.E., Hogquist K.A., Jameson S.C. (2016). IL-4 sensitivity shapes the peripheral CD8+ T cell pool and response to infection. J. Exp. Med..

[7] Baez N.S., Cerban F., Savid-Frontera C., Hodge D.L., Tosello J., Acosta-Rodriguez E., Almada L., Gruppi A., Viano M.E., Young H.A. (2019). Thymic expression of IL-4 and IL-15 after systemic inflammatory or infectious Th1 disease processes induce the acquisition of “innate” characteristics during CD8+ T cell development. PLoS Pathog..

[8] Shi X., Guo L.W., Seedial S.M., Si Y., Wang B., Takayama T., Suwanabol P.A., Ghosh S., DiRenzo D., Liu B. (2014). TGF-beta/Smad3 inhibit vascular smooth muscle cell apoptosis through an autocrine signaling mechanism involving VEGF-A. Cell Death Dis..

[9] Huang W., Huang F., Kannan A.K., Hu J., August A. (2014). ITK tunes IL-4-induced development of innate memory CD8+ T cells in a gammadelta T and invariant NKT cell-independent manner. J. Leukoc. Biol..

[10] Nayar R., Enos M., Prince A., Shin H., Hemmers S., Jiang J.K., Klein U., Thomas C.J., Berg L.J. (2012). TCR signaling via Tec kinase ITK and interferon regulatory factor 4 (IRF4) regulates CD8+ T-cell differentiation. Proc. Natl. Acad. Sci. USA.

[11] Lee Y.J., Holzapfel K.L., Zhu J., Jameson S.C., Hogquist K.A. (2013). Steady-state production of IL-4 modulates immunity in mouse strains and is determined by lineage diversity of iNKT cells. Nat. Immunol..

[12] Lai D., Zhu J., Wang T., Hu-Li J., Terabe M., Berzofsky J.A., Clayberger C., Krensky A.M. (2011). KLF13 sustains thymic memory-like CD8(+) T cells in BALB/c mice by regulating IL-4-generating invariant natural killer T cells. J. Exp. Med..

[13] Waickman A.T., Park J.Y., Park J.H. (2016). The common gamma-chain cytokine receptor: Tricks-and-treats for T cells. Cell. Mol. Life Sci..

[14] Ivashkiv L.B. (2018). IFNgamma: Signalling, epigenetics and roles in immunity, metabolism, disease and cancer immunotherapy. Nat. Rev. Immunol..

[15] Won H.Y., Kim H.K., Crossman A., Awasthi P., Gress R.E., Park J.H. (2021). The Timing and Abundance of IL-2Rbeta (CD122) Expression Control Thymic iNKT Cell Generation and NKT1 Subset Differentiation. Front. Immunol..

[16] Dalton D.K., Pitts-Meek S., Keshav S., Figari I.S., Bradley A., Stewart T.A. (1993). Multiple defects of immune cell function in mice with disrupted interferon-gamma genes. Science.

[17] Park J.Y., Won H.Y., DiPalma D.T., Kim H.K., Kim T.H., Li C., Sato N., Hong C., Abraham N., Gress R.E. (2022). In vivo availability of the cytokine IL-7 constrains the survival and homeostasis of peripheral iNKT cells. Cell Rep..

[18] Prakhar P., Alvarez-DelValle J., Keller H., Crossman A., Tai X., Park Y.K., Park J.H. (2021). The small intestine epithelium exempts Foxp3+ Tregs from their IL-2 requirement for homeostasis and effector function. JCI Insight.

[19] Morris S.C., Heidorn S.M., Herbert D.R., Perkins C., Hildeman D.A., Khodoun M.V., Finkelman F.D. (2009). Endogenously produced IL-4 nonredundantly stimulates CD8+ T cell proliferation. J. Immunol..

[20] Li C., Park J.H. (2020). Assessing IL-2-Induced STAT5 Phosphorylation in Fixed, Permeabilized Foxp3(+) Treg Cells by Multiparameter Flow Cytometry. STAR Protoc..

[21] Yarilin A.A., Belyakov I.M. (2004). Cytokines in the thymus: Production and biological effects. Curr. Med. Chem..

[22] Do J.S., Fink P.J., Li L., Spolski R., Robinson J., Leonard W.J., Letterio J.J., Min B. (2010). Cutting edge: Spontaneous development of IL-17-producing gamma delta T cells in the thymus occurs via a TGF-beta 1-dependent mechanism. J. Immunol..

[23] Lee Y.J., Jameson S.C., Hogquist K.A. (2011). Alternative memory in the CD8 T cell lineage. Trends Immunol..

[24] Verykokakis M., Boos M.D., Bendelac A., Kee B.L. (2010). SAP protein-dependent natural killer T-like cells regulate the development of CD8(+) T cells with innate lymphocyte characteristics. Immunity.

[25] Oghumu S., Terrazas C.A., Varikuti S., Kimble J., Vadia S., Yu L., Seveau S., Satoskar A.R. (2015). CXCR3 expression defines a novel subset of innate CD8+ T cells that enhance immunity against bacterial infection and cancer upon stimulation with IL-15. FASEB J..

[26] Renz H., Domenico J., Gelfand E.W. (1991). IL-4-dependent up-regulation of IL-4 receptor expression in murine T and B cells. J. Immunol..

[27] Leonard W.J., Lin J.X., O’Shea J.J. (2019). The γ(c) Family of Cytokines: Basic Biology to Therapeutic Ramifications. Immunity.

[28] Mathieu C., Beltra J.C., Charpentier T., Bourbonnais S., Di Santo J.P., Lamarre A., Decaluwe H. (2015). IL-2 and IL-15 regulate CD8+ memory T-cell differentiation but are dispensable for protective recall responses. Eur. J. Immunol..

[29] Mitchell D.M., Ravkov E.V., Williams M.A. (2010). Distinct roles for IL-2 and IL-15 in the differentiation and survival of CD8+ effector and memory T cells. J. Immunol..

[30] Mendoza J.L., Escalante N.K., Jude K.M., Sotolongo Bellon J., Su L., Horton T.M., Tsutsumi N., Berardinelli S.J., Haltiwanger R.S., Piehler J. (2019). Structure of the IFNγ receptor complex guides design of biased agonists. Nature.

[31] Kwesi-Maliepaard E.M., Jacobs H., van Leeuwen F. (2021). Signals for antigen-independent differentiation of memory CD8(+) T cells. Cell. Mol. Life Sci..

[32] Smith N.L., Patel R.K., Reynaldi A., Grenier J.K., Wang J., Watson N.B., Nzingha K., Yee Mon K.J., Peng S.A., Grimson A. (2018). Developmental Origin Governs CD8(+) T Cell Fate Decisions during Infection. Cell.

[33] Hussain T., Quinn K.M. (2019). Similar but different: Virtual memory CD8 T cells as a memory-like cell population. Immunol. Cell Biol..

[34] Tan J.T., Ernst B., Kieper W.C., LeRoy E., Sprent J., Surh C.D. (2002). Interleukin (IL)-15 and IL-7 jointly regulate homeostatic proliferation of memory phenotype CD8+ cells but are not required for memory phenotype CD4+ cells. J. Exp. Med..

[35] Istaces N., Splittgerber M., Lima Silva V., Nguyen M., Thomas S., Le A., Achouri Y., Calonne E., Defrance M., Fuks F. (2019). EOMES interacts with RUNX3 and BRG1 to promote innate memory cell formation through epigenetic reprogramming. Nat. Commun..

[36] Bortoluzzi S., Dashtsoodol N., Engleitner T., Drees C., Helmrath S., Mir J., Toska A., Flossdorf M., Ollinger R., Solovey M. (2021). Brief homogeneous TCR signals instruct common iNKT progenitors whose effector diversification is characterized by subsequent cytokine signaling. Immunity.

[37] Gordy L.E., Bezbradica J.S., Flyak A.I., Spencer C.T., Dunkle A., Sun J., Stanic A.K., Boothby M.R., He Y.W., Zhao Z. (2011). IL-15 regulates homeostasis and terminal maturation of NKT cells. J. Immunol..

[38] Havenar-Daughton C., Li S., Benlagha K., Marie J.C. (2012). Development and function of murine RORγt+ iNKT cells are under TGF-beta signaling control. Blood.

[39] Watarai H., Sekine-Kondo E., Shigeura T., Motomura Y., Yasuda T., Satoh R., Yoshida H., Kubo M., Kawamoto H., Koseki H. (2012). Development and function of invariant natural killer T cells producing T(h)2- and T(h)17-cytokines. PLoS Biol..

[40] Hogquist K., Georgiev H. (2020). Recent advances in iNKT cell development. F1000Res.

[41] Stock P., Lombardi V., Kohlrautz V., Akbari O. (2009). Induction of airway hyperreactivity by IL-25 is dependent on a subset of invariant NKT cells expressing IL-17RB. J. Immunol..

