# Supplementary material for: Proinflammatory IFNγ Is Produced by but Not Required for the Generation of Eomes+ Thymic Innate CD8 T Cells
Source: Cells. 2023 Oct 11;12(20):2433. doi: 10.3390/cells12202433 (PMC10605631; doi:10.3390/cells12202433)
Supplement: Supplementary file 1 [file cells-12-02433-s001.zip › cells-2571401-supplementary.pdf]

Figure S1

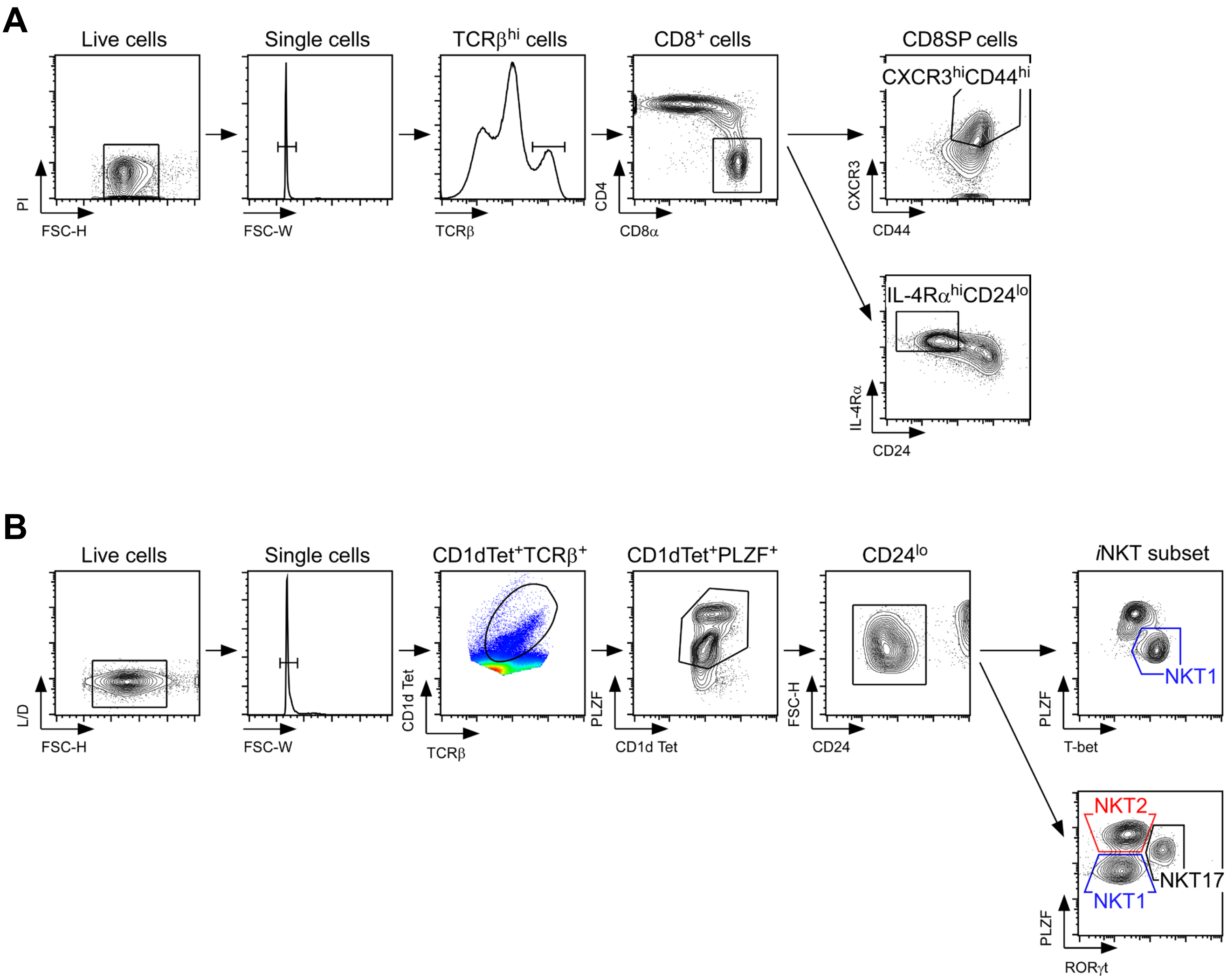

Figure S1. Representative gating strategies for thymic innate CD8 T cells and *i*NKT subsets
All flow cytometric analyses were performed after live cell gating based on forward size scatter (FSC-H) and exclusion of dead cells either by propidium iodide counter staining for fresh cells **(A)** or Ghost Dye (Tonbo Bioscience) for fixed cells **(B)**. Innate CD8 T cells were identified as CXCR3^{hi}CD44^{hi} or IL-4Rα^{hi}CD24^{lo} cells among CD8 single positive TCRβ high expressers **(A)**. *i*NKT cell subsets were identified by gating on TCRβ^{+}CD1dTet^{+} cells followed by PLZF^{+}CD1dTet^{+} gating. Mature iNKT cells were then identified as CD24^{lo} cells, which were then further plotted for T-bet versus PLZF or RORγt versus PLZF expression **(B)**.

Figure S2

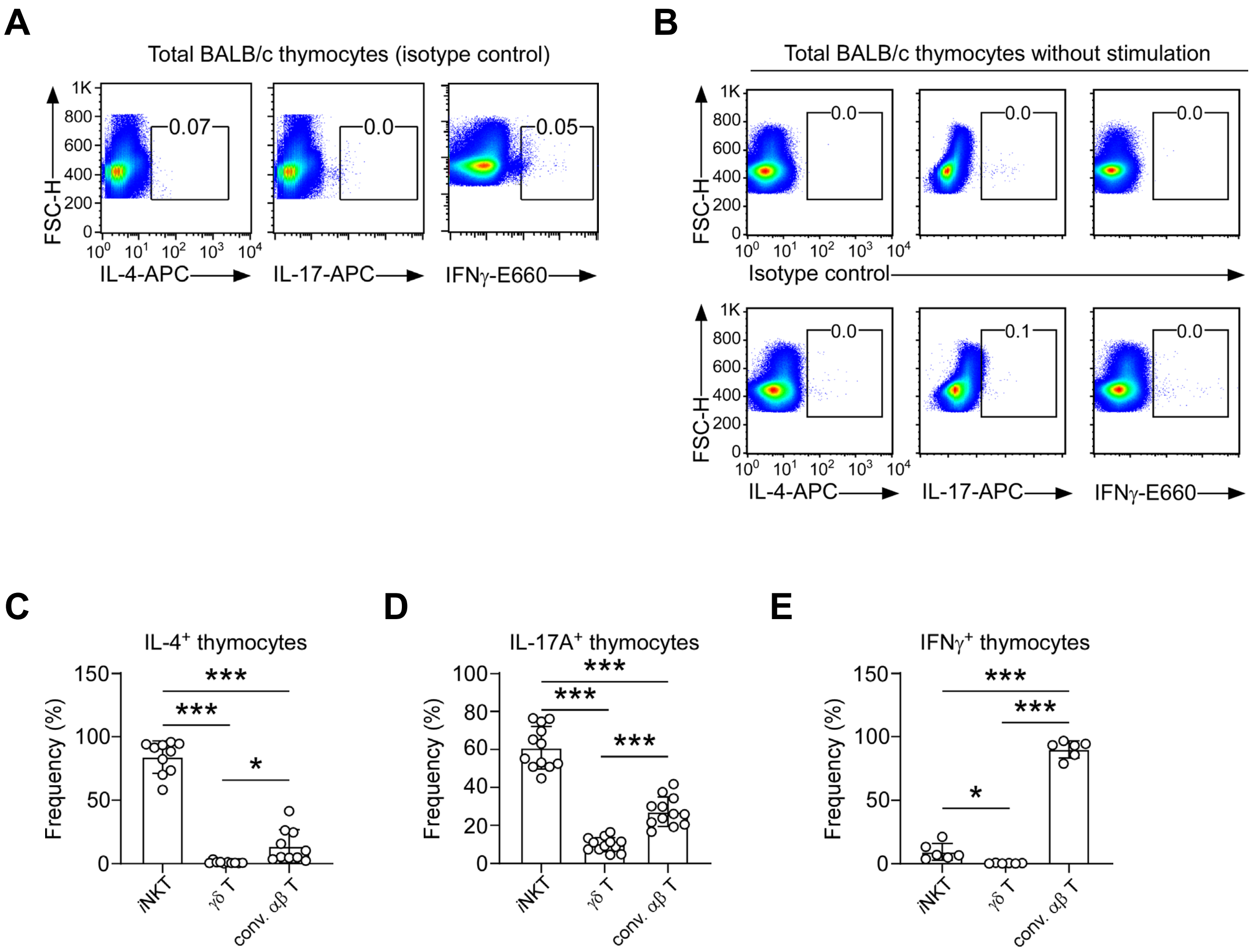

Figure S2. T cell subset distribution of cytokine-producing BALB/c thymocytes
(A) Total thymocytes of BALB/c mice were incubated *in vitro* with PMA, Ionomycin and BFA for 4 hours. The contour plots show the isotype control of intracellular IL-4 (left), IL-17 (middle) and IFNγ (right) staining.
(B) Total thymocytes of BALB/c mice were incubated *in vitro* with BFA for 4 hours without any stimulation. The contour plots show the isotype control on top and intracellular IL-4 (left), IL-17 (middle) and IFNγ (right) staining on bottom.
(C) Frequency of *i*NKT, γδ T, and conventional αβ T cells among IL-4-producing thymocytes in BALB/c mice. Data show summary of 3 independent experiments with 10 BALB/c mice.
(D) Frequency of *i*NKT, γδ T, and conventional αβ T cells among IL-17A-producing thymocytes in BALB/c mice. Data show summary of 4 independent experiments with 12 BALB/c mice.
(E) Frequency of *i*NKT, γδ T, and conventional αβ T cells among IFNγ-producing thymocytes in BALB/c mice. Data show summary of 2 independent experiments with a total of 6 BALB/c mice.

Figure S3

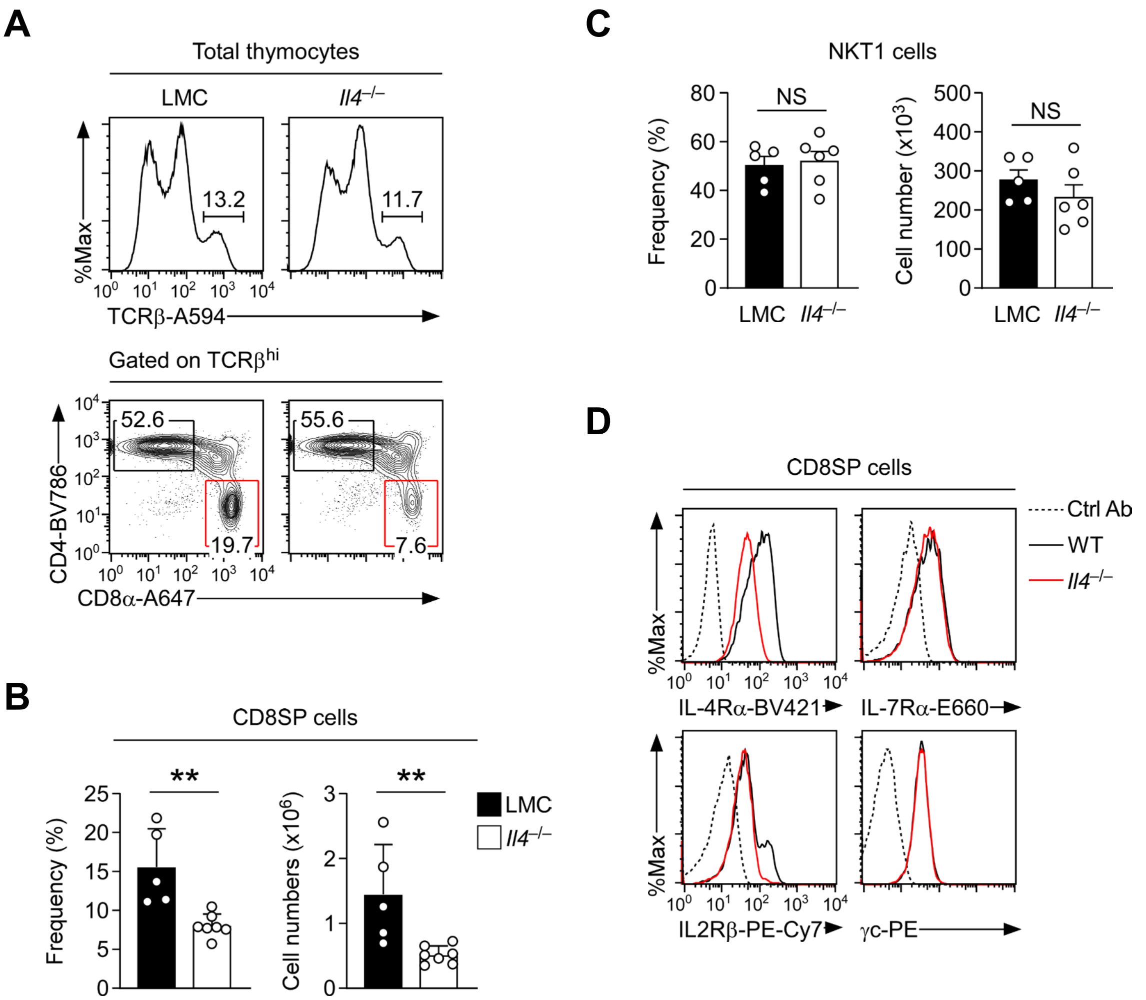

Figure S3. CD8 T cell development in *Il4*–/– BALB/c mice

(A) Mature CD4SP and CD8SP cells (bottom) were identified among TCRβ^{hi} mature thymocytes of *Il4*–/– and LMC BALB/c mice (top).

(B) Frequency and number of mature CD8SP thymocytes in *Il4*–/– and LMC BALB/c mice. Data show summary of 4 independent experiments with 7 *Il4*–/– and 5 LMC BALB/c mice.

(C) Frequency and number of thymic NKT1 cells in *Il4*–/– and LMC BALB/c mice. Results are the summary of 3 independent experiments with a total of 6 *Il4*–/– and 5 LMC BALB/c mice.

(D) Cytokine receptor expression on TCRβ^{hi} mature CD8SP thymocytes of LMC and *Il4*–/– BALB/c mice. Results are representative of 2 independent experiments.

Figure S4

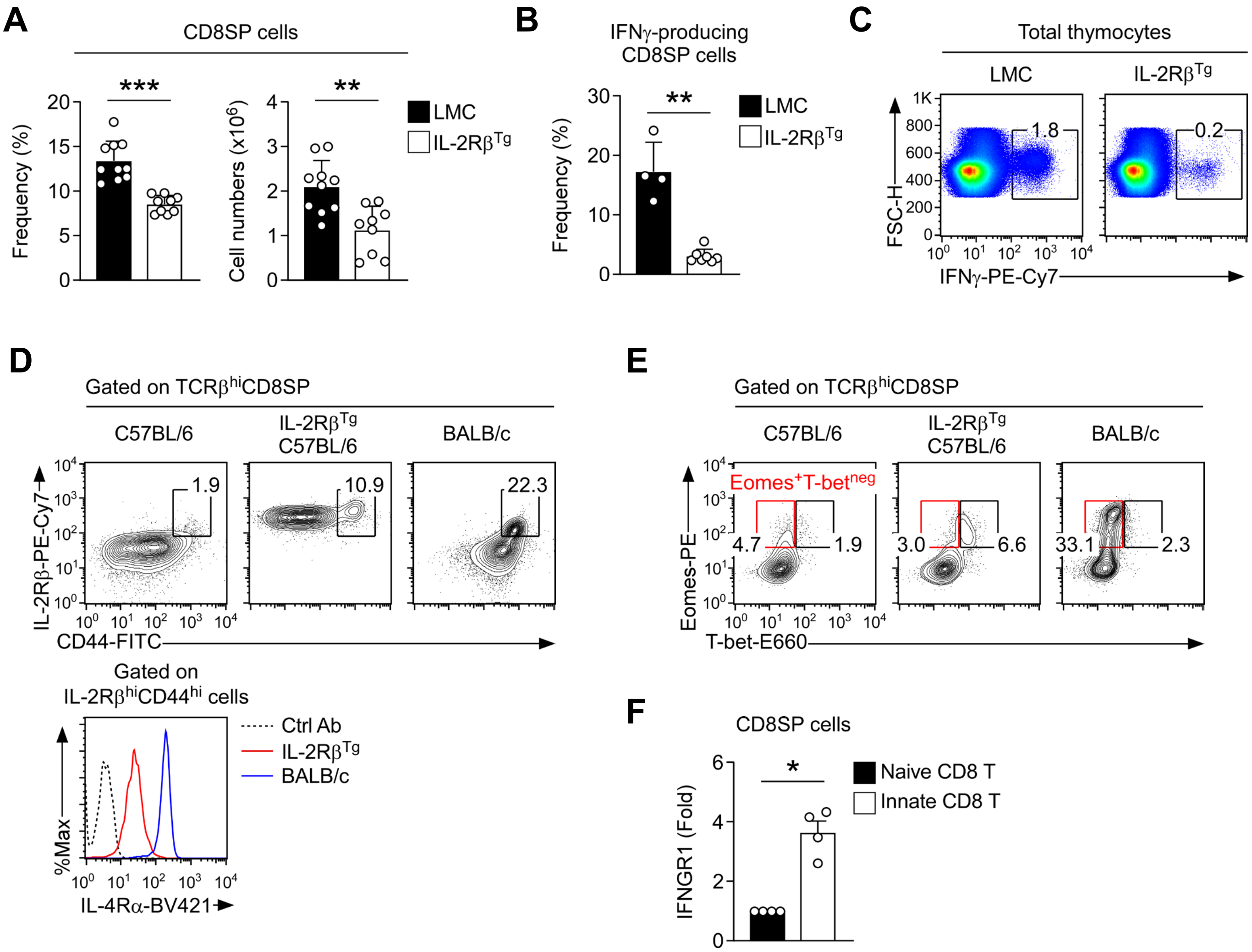

Figure S4. CD8 T cell development in IL-2Rβ^{Tg} BALB/c mice

(A) Frequency and number of mature CD8SP thymocytes in IL-2Rβ^{Tg} and LMC BALB/c mice. Data show summary of 6 independent experiments with 9 IL-2Rβ^{Tg} and 10 LMC BALB/c mice.

(B) Frequency of IFNγ^{+} cells among CD8SP thymocytes of IL-2Rβ^{Tg} and LMC BALB/c mice. Data show summary of 3 independent experiments with 7 IL-2Rβ^{Tg} and 4 LMC BALB/c mice.

(C) Frequency of IFNγ^{+} thymocytes of IL-2Rβ^{Tg} and LMC BALB/c mice. Results are representative of 3 independent experiments with a total of 5 IL-2Rβ^{Tg} and 5 LMC BALB/c mice.

(D) IL-2Rβ^{Tg} mice on C57BL/6 background were assessed for the presence of IL-2Rβ^{hi}CD44^{hi} memory phenotype CD8SP thymocytes (top) and their surface IL-4Rα expression (bottom). Results are representative of **3** independent experiments with a total of 3 C57BL/6, 5 IL-2Rβ C57BL/6 and 3 BALB/c mice.

(E) Intranuclear Eomes versus T-bet expression in mature CD8SP thymocytes of the indicated mice. Cells in red boxes correspond to innate CD8 T cells. Results are representative of 3 independent experiments.

(F) INFGR1 expression on naïve (IL-4Rα^{lo}CD24^{hi}) and innate (IL-4Rα^{hi}CD24^{lo}) CD8SP BALB/c thymocytes. Bar graphs show the summary of 2 independent experiments with 4 BALB/c mice.

Figure S5

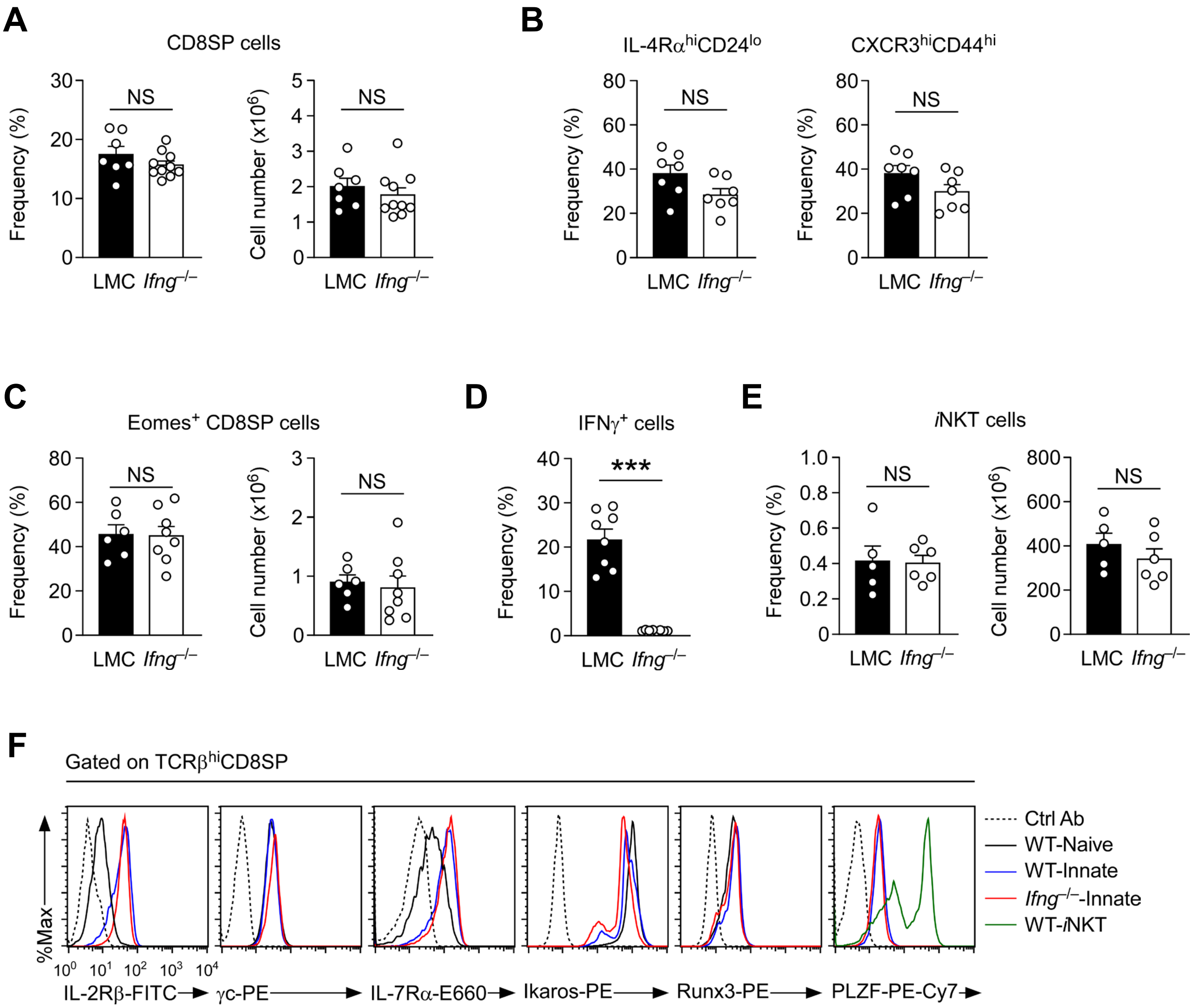

Figure S5. Thymic innate CD8 T cell generation in IFNγ-deficient mice
Frequencies and numbers of mature CD8SP cells **(A)**, IL-4Rα^{hi}CD24^{lo} and CXCR3^{hi}CD44^{hi} CD8SP cells **(B)** and intranuclear Eomes^{+} CD8SP cells **(C)** in LMC and *Ifng*$^{-/-}$ BALB/c mice. Data are the summaries of at least 3 independent experiments with at least 6 LMC and 7 *Ifng*$^{-/-}$ BALB/c mice.
(D) Frequencies of IFNγ-producing cells among mature CD8SP thymocytes. Results are a summary of 3 independent experiments with each 8 LMC and 8 *Ifng*$^{-/-}$ BALB/c mice.
(E) Frequencies and numbers of thymic *i*NKT cells in LMC and *Ifng*$^{-/-}$ BALB/c mice. Data are representative of 3 independent experiments with a total of 5 LMC and 6 *Ifng*$^{-/-}$ BALB/c mice.
(F) Histograms show the expression of innate CD8 T cell-associated markers in mature CD8SP thymocytes of LMC and *Ifng*$^{-/-}$ BALB/c mice. Data are representative of 2 independent experiments with a total of 4 LMC and 5 *Ifng*$^{-/-}$ BALB/c mice. NS, not significant, *** $p \leq 0.0005$

Figure S6

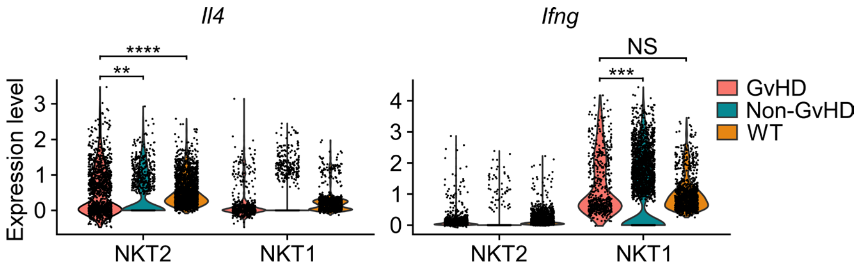

Figure S6. IL-4 and IFNγ expression in NKT1 and NKT2 cells
Violin plots show single cell expression distributions of IL-4 and IFNγ in the cluster NKT1 and NKT2. Single-cell RNA-seq data (GSE228645) were downloaded from GEO database and reanalyzed in R language using "Seurat" package. Statistic significance among groups was calculated by using "ggpubr" package. GvHD, bone marrow transplantation (BMT) mice with chronic graft-versus-host disease; Non-GvHD, BMT mice without GvHD; WT, C57BL/6 mice without BMT. NS, $p > 0.05$; ** $p \leq 0.05$; *** $p \leq 0.001$; **** $p \leq 0.0001$;

Figure S7

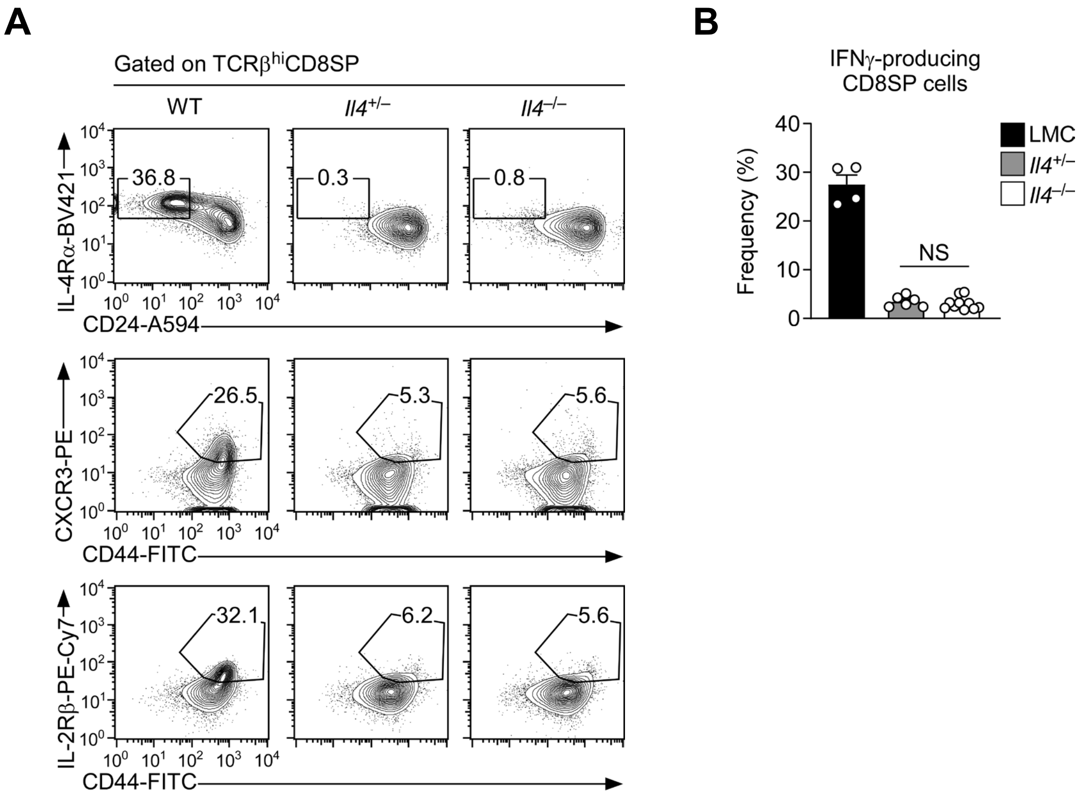

Figure S7. Innate CD8 T cells and thymic *i*NKT cells of IL-4-deficient mice

(A) Innate CD8 T cell frequencies as determined by IL-4Rα versus CD24, CXCR3 versus CD44, and IL-2Rβ versus CD44 staining of CD8SP thymocytes of *Il4*$^{-/-}$, *Il4*$^{+/-}$, and LMC BALB/c mice. Results are representative of 4 independent experiments.

(B) Frequency of IFNγ^{+} cells among CD8SP thymocytes of *Il4*$^{-/-}$, *Il4*$^{+/-}$, and LMC BALB/c mice. Data show summary of 4 independent experiments with 10 *Il4*$^{-/-}$, 6 *Il4*$^{+/-}$, and 4 LMC BALB/c mice.

Figure S8

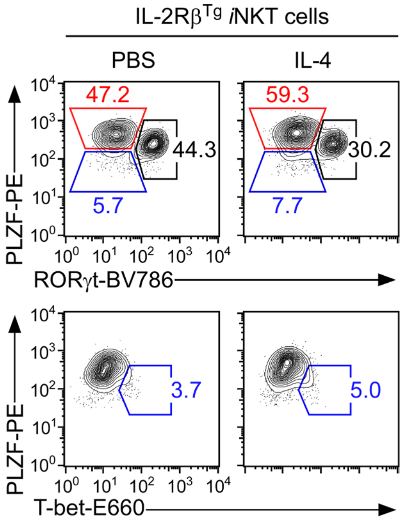

Figure S8. Thymic *i*NKT cells of IL-4-treated IL-2Rβ^{Tg} BALB/c mice
Thymic *i*NKT subset compositions were assessed in CD24^{lo} mature *i*NKT cells by intracellular RORγt versus PLZF (top) and T-bet versus PLZF staining (bottom). Results are representative of 2 independent experiments with a total of 5 IL-2Rβ^{Tg} BALB/c mice that were injected with PBS or recombinant IL-4 and anti-IL-4 antibodies.
